# Approaches to Evaluate the Impact of Community-Based Delivery Strategies

**DOI:** 10.4269/ajtmh.16-0110a

**Published:** 2016-06-01

**Authors:** Tanya Doherty, Kate Kerber, Mary Kinney, John Mason

**Affiliations:** Health Systems Research Unit, South African Medical Research Council, School of Public Health, University of the Western Cape, Cape Town, South Africa. E-mail: tanya.doherty@mrc.ac.za; Save the Children, Washington, DC. E-mails: kkerber@savechildren.org and mkinney@savechildren.org; Department of Global Community Health and Behavioral Sciences, Tulane School of Public Health and Tropical Medicine, New Orleans, LA. E-mail: johnbmason2@gmail.com

Dear Sir:

The article by Amouzou and others^1^ presents findings from an evaluation of integrated community case management (iCCM) in Malawi using a National Evaluation Platform (NEP) design. While it is a welcome effort to document this child survival strategy that is being tested in many low- and middle-income countries, the study findings have implications both for iCCM and for approaches to evaluation, which would benefit from further scrutiny.

An important consideration in the evaluation of community-based care is ascertaining true exposure to the intervention in the study population. The article by Amouzou and others used a dose–response approach, with 27 districts as the unit of analysis, to measure the effect of an intervention (iCCM) that was specifically targeted at hard-to-reach areas. Their approach ignored spatial variation within districts and led to a diluted estimate of the effect of iCCM, as not all communities sampled in the household surveys were exposed to iCCM-trained health surveillance assistants (HSAs). A more effective approach would have been to stratify the samples by a measure of spatial exposure, for instance average travel time to the nearest HSA and ratio of HSAs per population within a given buffer of the cluster.^2^ Moreover, the exposure was limited at 1.5 HSAs per 1,000 children under 5 years of age (lower than the Ministry of Health target of one per 1,000 population, equivalent to about one HSA per 200 children). For community-based nutrition programs, a ratio of one community worker per 100–200 children is associated with impact on child malnutrition.^3^

Furthermore, the regression analysis used overall care seeking from all formal providers as the dependent variable, not care seeking from HSAs who actually delivered iCCM. A more accurate approach would have been to use change in care seeking to HSAs as the dependent variable. In addition, care seeking does not necessarily result in appropriate treatment. Therefore, it may be misleading to conclude that iCCM had no impact on under-five mortality without assessing changes in coverage of appropriate treatment of childhood illnesses.

As more countries are scaling up community-based delivery platforms,^4^ it is critical that methods to evaluate the impact of these services take into consideration spatial exposure to community health workers. National household surveys, which are the main source of exposure and outcome data in the NEP design, are designed to provide estimates at the district or regional level, and therefore cannot provide valid estimates among populations exposed to iCCM when exposure varies within the district or region. We agree with the authors' request for more investigation into why about one-third of HSAs do not live in the community they serve and how this may affect access for sick children.^5^

Given the considerations presented, we question the validity of the conclusion that iCCM had no impact on care seeking or mortality in Malawi. An alternative interpretation could be that at this exposure, the effects were too small to establish with the methods used; an estimate of the possible effect size, rather than negative conclusions, would be more useful. There are important policy and programmatic risks in concluding that iCCM has no impact. Policy makers could change the course of programs or plans, donors could shift priorities, and program managers could be demotivated by a sense of failure. Thus, there is need for caution when interpreting the findings. We support the authors' call for continuous large-scale evaluation in global health, including rigorous methodology with appropriate evaluation designs, to provide robust conclusions of relevance to improving health services.
